# An Oncology NP-MD Partnership: Challenges and Rewards

**Published:** 2018-05-01

**Authors:** Catherine S. Bishop

**Affiliations:** Johns Hopkins Kimmel Cancer Center at Sibley Memorial Hospital, Washington, DC

## Abstract

The State of Cancer Care in America report shines a spotlight on the significant increase of cancer survivors by 2026. One strategy to help care for the increased number of patients that require ongoing care is to hire more advanced practice providers. This article provides some keys to developing a positive, professional partnership with oncologists.

The 2017 State of Cancer Care in America report by the American Society of Clinical Oncology highlights progress and opportunities in the ever-changing and challenging specialty of oncology. According to the report, the number of cancer survivors is predicted to reach 20.3 million by 2026. This is a 31% increase from 2016 (American Society of Clinical Oncology, 2017). Members of oncology organizations are thinking of strategies to manage the influx of patients in the coming years. Among the many strategies being considered is hiring more advanced practice providers. Many of those in oncology practices believe advanced practice providers enhance patient care (Bishop, 2009).

A collaborative practice between a nurse practitioner (NP) and a medical doctor (MD) in oncology provides the opportunity to enhance patient care, create consistency in cancer management, and increase patient satisfaction (Bishop, 2009). Collaboration is defined as a true partnership in which both parties value the unique role of each provider and share the common goal of quality patient care (Dougherty & Larson, 2005).

There are different NP-MD practice models utilized in oncology. The state scope of NP practice will often influence the type of model used (Table 1 and Figure 1). However, there are other variables that may guide practitioners to utilize one model over the next.

**Table 1:**
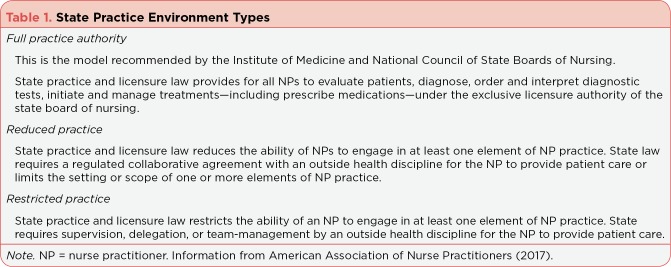
State Practice Environment Types

**Figure 1:**
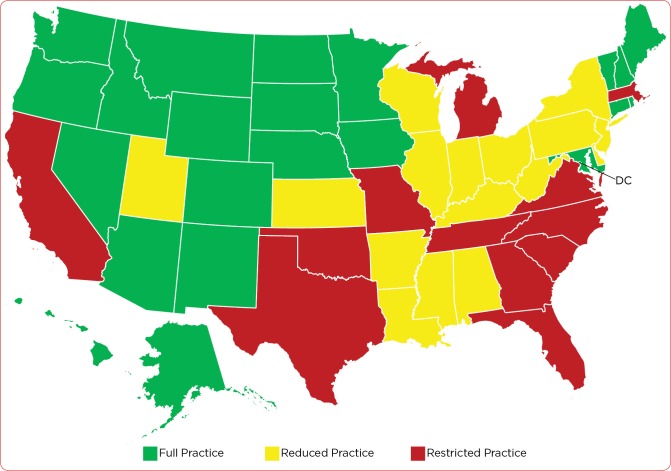
Nurse Practitioner Full Practice Authority States and District of Columbia

## NP SCOPE OF PRACTICE VARIES BY STATE

Nurse practice laws and regulations are specific to each state. There are three practice environment types: full practice authority (22 states and the District of Columbia have approved full practice authority; Table 2), reduced practice (states include Kansas, New Jersey, and New York), and restricted practice (states include Texas, Missouri, California, and Florida).

**Table 2:**
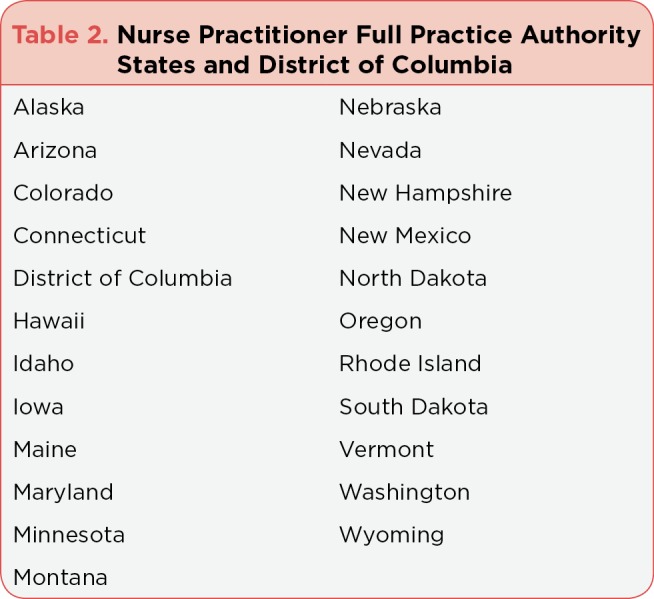
State Practice Environment Types

Full practice authority allows NPs to practice without supervision by a physician. Within the specific state scope of practice, there is no requirement for a collaborative agreement with a physician. Reduced practice reduces the ability of NPs to engage in at least one element of NP practice. A collaborative agreement is required and includes a designated physician collaborator(s). Restricted practice restricts the ability of NPs to engage in at least one element of their practice and requires supervision, delegation, or team management by a physician(s) in order for the NP to practice within that state (American Association of Nurse Practitioners, 2017).

Many NPs endorse full practice authority. Collaboration with our physician partners is a constant within this model. Collaborating with our physician partners as well as the other members of the health-care team is vital to safe and efficient cancer care. No one in oncology practices in a silo. Other variables that may guide practice models are NP level of experience, disease-specific knowledge, and style of each practitioner.

## FORMING A PARTNERSHIP

The creation of a professional, collaborative relationship between an oncology nurse practitioner and an oncologist is many times a dance in which both parties are either in rhythm, or sometimes out of step. As with most relationships, it takes time, mutual respect, patience, trust, and chemistry to make the partnership work well for both the providers and patients. Bedside manner matters. It is important that each provider has a similar patient care philosophy if the partnership is to work. Cancer management philosophy is critical when sharing the patient population, and patients must feel that both providers are in concert when providing their care. This requires consistent and clear communication between the NP and the MD. It is critical to allow for some professional disagreement that ultimately leads to the most satisfying outcome for the patient. Collaborative communication has been defined as both disciplines working in a cooperative manner and creating an environment that fosters conflict management, problem solving, and shared decision-making to achieve the goal of promoting excellence in patient outcomes (Boyle & Kochinda, 2004).

My story is worth sharing for other potential partnerships. In 2014, our academic practice hired an oncologist Dr. K, with over 35 years of private practice experience. He joined our practice with vast experience in treating all solid and hematologic cancers. My background included many years in an academic cancer center, as well as private practice experience where I had great physician mentors. I had the privilege of managing a variety of different cancers, as well as performing bone marrow biopsies and other procedures.

When Dr. K joined our practice in 2014, he brought a large portion of his former private practice’s patient population with him. It was clear that in the new environment where the processes and office staff were all new to him, he needed a partner to help care for the influx of patients and the many responsibilities that are intertwined in caring for each patient. With my varied background and experience, the best person to fit into the position was me. We joined hands (so to speak) and began the dance.

## PATIENT COMMUNICATION

The first item on the agenda was to create a letter that would be sent to all his former patients who had followed him to our practice—many of whom had been with him for several years and were very accustomed to only seeing Dr. K. Both Dr. K and I had input regarding the letter. The objective of the letter was to advocate for the collaborative partnership and define our commitment to work together for each patient in a team-like atmosphere. The letter was as follows:

"*Dear Patient:*

*Working together with my new Hopkins colleagues, we are committed to providing you with the highest quality cancer care in the capital region.*

*In furthering that commitment, I would like to introduce you to Catherine Bishop, DNP, NP, AOCNPィ, who will be partnering with me in your care moving forward. Dr. Bishop holds a Doctorate in Nursing Practice with certification as an Advanced Oncology Nurse Practitioner.*

*We have combined our practices to best serve you and intend to work as a seamless team through our close and frequent communication, particularly when changes in a patient’s status occur. Dr. Bishop has decades of experience at George Washington University Cancer Center and in private oncology practices. She has published articles in several peer-reviewed journals on topics such as breast cancer and hematologic malignancies. Most importantly, she has my full confidence, and I am sure will earn yours.*"

This letter was critical to a successful transition from seeing only Dr. K to seeing me as well. The communication paved the way for our clinic schedulers to offer the next visit with me. There were challenges with some patients, but most were accepting. Going forward, each time a patient had a visit with Dr. K, he indicated that he would like him or her to see me next. My schedule grew quickly. It was imperative that Dr. K be my best advocate if the partnership was to be successful. Our practice evolved into an alternating visit format. The alternating visits allowed for continuity of care, and most patients seemed to enjoy the new partnership. I would often tell patients they had two brains and four eyes looking out for them. Patients were reassured knowing that both Dr. K and I were involved in their cancer care.

## TEAM MEETINGS

The second item on the agenda was for us to implement daily meetings to review and discuss all of the patients on our respective schedules. We have found that a convenient time for us is every evening after clinic. In these meetings, we review all patients on my schedule for the following day. This discussion allows for uninterrupted time to learn about patients I have never seen before but will see the next day, or patients Dr. K saw during the previous visit. Additionally, the discussion includes patients on his schedule from that day, including new patients who need chemotherapy orders submitted, new consults, or patients requiring bone marrow biopsy. We often discuss results of imaging studies, alternative chemotherapy regimens for patients who have progressed, next steps for patients with rising tumor markers, and new drugs with recent approval or clinical trials in which patients may be eligible candidates for in the future. This format provides for continuity of care throughout the patients’ cancer journeys. Additionally, it reassures our patients to know that we consistently communicate regarding their treatment and overall cancer management.

We discuss new patients requiring chemotherapy and share in the effort of writing chemotherapy orders in our electronic ordering system. Within our institution, oncology NPs must meet certain criteria in order to create the chemotherapy treatment plan. We also share in performing bone marrow biopsy/aspiration procedures. The partnership has been an effective one for patients and for us. Four years later, we have successfully mastered the dance and appear to be in step with one another.

## FINAL THOUGHTS

Much is to be learned in oncology today, and keeping pace with the ever-constant new drug approvals and scientific advances is challenging. It takes a team to care for each individual patient, and I feel fortunate to be part of a team steeped in respect and trust. My partnership includes not only Dr. K, but also the dedicated and thoughtful oncology nurses, medical assistants, oncology social work professionals, and administrative staff members who are on our team. We all work in concert to provide the best care possible to each patient.
